# Association between use of psychotropic medications prior to SARS-COV-2 infection and trajectories of COVID-19 recovery: Findings from the prospective Predi-COVID cohort study

**DOI:** 10.3389/fpubh.2023.1055440

**Published:** 2023-03-16

**Authors:** Gloria A. Aguayo, Aurélie Fischer, Abir Elbéji, Nyan Linn, Markus Ollert, Guy Fagherazzi

**Affiliations:** ^1^Deep Digital Phenotyping Research Unit, Department of Precision Health, Luxembourg Institute of Health, Strassen, Luxembourg; ^2^UNAIDS, Yangon, Myanmar; ^3^Allergy and Clinical Immunology, Department of Infection and Immunity, Luxembourg Institute of Health, Strassen, Luxembourg

**Keywords:** latent class trajectory analyses, symptom score, psychotropic medication, depressive symptoms, anxiety, mental health, COVID-19, cohort study (or longitudinal study)

## Abstract

Psychological disturbances are frequent following COVID-19. However, there is not much information about whether pre-existing psychological disorders are associated with the severity and evolution of COVID-19. We aimed to explore the associations between regular psychotropic medication use (PM) before infection as a proxy for mood or anxiety disorders with COVID-19 recovery trajectories. We used data from the Predi-COVID study. We followed adults, tested positive for SARS-CoV-2 and collected demographics, clinical characteristics, comorbidities and daily symptoms 14 days after inclusion. We calculated a score based on 16 symptoms and modeled latent class trajectories. We performed polynomial logistic regression with PM as primary exposure and the different trajectories as outcome. We included 791 participants, 51% were men, and 5.3% reported regular PM before infection. We identified four trajectories characterizing recovery dynamics: “Almost asymptomatic,” “Quick recovery,” “Slow recovery,” and “Persisting symptoms“. With a fully adjusted model for age, sex, socioeconomic, lifestyle and comorbidity, we observed associations between PM with the risks of being in more severe trajectories than “Almost Asymptomatic”: “Quick recovery” (relative risk (95% confidence intervals) 3.1 (2.7, 3.4), “Slow recovery” 5.2 (3.0, 9.2), and “Persisting symptoms“11.7 (6.9, 19.6) trajectories. We observed a gradient of risk between PM before the infection and the risk of slow or no recovery in the first 14 days. These results suggest that a pre-existing psychological condition increases the risk of a poorer evolution of COVID-19 and may increase the risk of Long COVID. Our findings can help to personalize the care of people with COVID-19.

## 1. Introduction

The severity of COVID-19 is heterogeneous and can range from asymptomatic to extreme severity and death (1). During the first weeks of the infection, the COVID-19 disease often presents clinically with mild symptoms and recovery at the end of the second week. However, the severity of COVID-19 may vary in some people with aggravation or persistent symptoms, now known as long COVID (2). A study that analyzed the acute symptoms reported that specific symptoms such as fatigue might predict hospital care and respiratory support (3). A cohort study showed that individuals older than 65 had a higher risk of persistent symptoms after the acute Covid infection phase, such as respiratory insufficiency, hypertension, kidney problems, memory complaints and mental health conditions (4).

People with a more severe gravity at an early stage have a higher risk of developing chronic symptomatology in the long term (5). Therefore, early symptoms are essential to predict future long COVID: more than five symptoms during the first week of infection is associated with a higher risk of developing long COVID (6). Galal et al. created a symptom score that, at the acute stage, was correlated with long COVID symptoms (7). Using a score of symptoms can help analyze the severity of the disease.

The COVID-19 pandemic had a significant impact on the mental health of the population (8, 9). There is evidence of the impact of COVID-19 symptoms on short and long-term psychological symptoms (10, 11). A recent study revealed the association between psychological distress and concomitant COVID-19 symptoms (12). Moreover, there is some evidence of the effect of depression on immunity (13). While there is much evidence of how COVID-19 infection and lockdown influence mental health trajectories, there is evidence of a higher risk of COVID-19 infection in people with pre-existing psychological comorbidity (14). However, it is unknown whether people with psychological disorders could evolve differently concerning COVID-19 symptoms.

Therefore, the objective of this study was to analyze the role of anxiety or mood disorders as a determinant of COVID-19 symptom trajectories.

## 2. Method

### 2.1. Participants

The Predi-COVID study is an ongoing hybrid cohort of people with confirmed SARS-CoV-2 infection. Participants were invited to Predi-COVID if they were SARS-CoV-2 positive and older than 18 years old. Inclusion was performed at the time of acute illness, at the hospital or home, either with or without symptoms, between May 2020 and June 2022. Due to the unknown about the spread of this pandemic, the sample size was not determined *a priori*. The baseline assessment consisted of data collected *via* phone calls and online questionnaires about demographics, epidemiological factors, lifestyle, comorbidity, and biomarkers. In addition, there were questions about medications, including the use of psychotropic medications. Then, there were daily questionnaires for 14 days about general health status and COVID-19-related symptoms. More details about the study are described elsewhere (15). The National Research Ethics Committee approved the study. All participants signed informed consent.

#### 2.1.1. Inclusion criteria

We included adult participants from the study with a positive PCR who had completed the baseline questionnaires and information about each medication they took regularly and had participated in at least one out of the14 days of the daily assessment.

### 2.2. Study design

This study is a secondary data analysis of the Predi-COVID study. It is a longitudinal latent class trajectory analysis with a follow-up of 14 days.

### 2.3. Outcome and main exposure

The outcome was 14-day trajectories of a total number of COVID-19-related symptoms. Filling an e-questionnaire proposed daily for 14 days after baseline, the participants answered questions about 16 symptoms (Supplementary Table 1). We then calculated a score representing the severity of the disease based on the 16 symptoms. The symptoms were: fatigue/feel bad, cough, cough aggravation, sore throat, loss of taste/smell, diarrhea, muscle aches, chest pain, pain scale, fever, difficulty breathing, increased breath difficulties, eating or drinking difficulties, skin rashes, conjunctivitis, and other symptoms. The fatigue/feel bad question had three possible answers: “I feel well,” “I feel fatigued/tired, and “I feel bad.” We assigned 0, 0.5 and 1 points, respectively. The pain scale asked to quantify pain chest from zero (no pain) to 10 (maximal pain). The values <2, ≥2 and < 3, ≥3 were recoded to 0, 0.5 and 1, respectively. For the 14 questions left, the possible answers were yes (reported symptom) or no (no reported symptom), and we assigned 1 and 0 values, respectively. The possible values of the score go from 0 points (no reported symptoms) to 16 points (all symptoms at a maximum value reported).

The primary exposure was the use of psychotropic medications (PM) at least three times a week before the COVID-19 diagnosis and assessed by a trained nurse during the inclusion phone call. The team checked the self-reported PM using information from each patient’s list of declared medications. It classified them into antidepressants, anxiolytics, anticonvulsants, hypnotics and antipsychotic medication using the Anatomical Therapeutic Chemical codes.

### 2.4. Covariates

We assessed demographic, psychosocial and comorbidity at baseline as possible determinants of latent classes. Age was analyzed continuously. Smoking status was categorical (current, former and never smoker). Education was categorized into low (only primary) education and medium-high (secondary school and above). Income was categorized into low income (lowest income tertile) and moderate-high income (second and third income tertile). Work status was classified as unemployed and employed with the question “Do you have a professional activity?”

BMI was calculated as weight/height^2^ (kg/m^2^) and categorized as obesity with a BMI ≥ 30 kg/m^2^ and no obesity with a BMI < 30 kg/m^2^. Physical activity was calculated as the average of usual winter and summer physical activity, including walking, cycling, gardening, cleaning and sport. It was categorized as low physical activity (first tertile) and moderate high physical activity (second and third tertile).

Diabetes was defined as a self-reported medical diagnosis or taking diabetes oral medication or insulin. Multimorbidity was defined as two or more chronic conditions among 16 conditions (self-reported hypertension, chronic heart disease, chronic lung disease, asthma, renal disease, moderate or severe liver disease, mild liver disease, chronic neurological disorder, cancer, chronic pulmonary disease, obesity, diabetes, rheumatic disease, malnutrition, COPD, other). Weight loss was defined as unintentional weight loss of 3 kg or more in the last 6 months before the COVID-19 infection. Polypharmacy was defined as taking two or more medications at least three times a week for any condition out of COVID-19.

### 2.5. Missing data

We assumed that missing data were missing at random. We described the percentage of missing data for each variable, and we applied multiple imputations to deal with missing data with the chained-equation approach (R package Mice) (16). The imputation model was performed by choosing the best predictors for missing data for each time point with the function “Quickpred” (17) and other relevant confounders and outcome variables. We imputed baseline predictors and missing values of symptoms for calculating scores. The symptom score was calculated from day 0 to day 14 *a posteriori* with the imputed symptom values in each imputed dataset. Then, we deleted the imputed values of scores of a day when the participant did not answer any of the questions about symptoms on that day. We generated 40 imputed datasets with 20 iterations. We tested the plausibility of imputed data with summary statistics.

### 2.6. Statistical analysis

We tested the distribution of continuous variables, and we described the numerical variables as mean (SD) when they were normally distributed and median (IQR) when they were not, and categorical variables with frequency (percentage).

We performed a latent class trajectory analysis (18) with one class, symptom trajectories as the outcome, and the day (ranging from 0 to 14) as the fixed and random effects. We tested four different structures assuming linearity or not: linear, non-linear Beta cumulative distribution function, non-linear Quadratic I-splines with five knots placed at quantiles of Y and non-linear I-splines with four equidistant nodes. We chose the model that had the lowest AIC. Then, we run seven models with the selected structure, each with one to seven classes. We applied the function grid and checked if the model achieved convergence. We chose the best model based on the following criteria the Bayesian Information Criterion (BIC) (the smallest the best), entropy (values from 0 to 1) should be equal or superior to 0.6 with each class should have at least 5% of the subjects (18, 19). We estimated for each individual the probability of correct classification with their posterior classification. We also described the baseline characteristics stratified by classes. We plotted the best model and then described the latent class associated with the lowest risk of disease severity.

We performed multinomial univariate logistic regression models. The trajectory of symptoms was the dependent variable, and PM was the determinant. We chose the class with the lowest trajectory regarding the number of symptoms at baseline as the reference. We progressively added confounders in the models. To be considered a confounder, the variable should be associated with the outcome symptom trajectory) and the exposure (psychological disturbances). Model 1 was adjusted for sex and age. Model 2 was further adjusted for work, income, smoking status, BMI, physical activity and multimorbidity. We did not include weight loss because we considered as a collider and polypharmacy was highly correlated with multimorbidity. We calculated the relative risk ratio as the exponentiated pooled coefficients of the imputed data sets and 95% confidence intervals according to Rubin’s rules. We used R Studio (R version 4.0.2) for all the analyzes, “lcmm” R package for trajectory analysis and “nnet” R package for multinomial analysis. We used an alpha <0.05 to define statistical significance.

Study method and results are reported following the “Strengthening the Reporting of Observational Studies in Epidemiology” in Supplementary material (STROBE) (20).

## 3. Results

There were 1,037 adult participants in this cohort study positive for SARS-CoV-2. We excluded 75 participants that did not provide baseline data. We further excluded and participated in the baseline questionnaires. Then, we excluded 171 participants who did not participate in any of the daily questionnaires. Finally, we analyzed 791 participants (Supplementary Figure S1). Missing data ranged from 0 to 44%. The mean (SD) age of the population was 40.0 (12.5) years; 403 (51%) were men. Forty-two participants (5.3%) reported PM. We found that people who reported PM also reported more baseline symptoms, current smoking, and multimorbidity. We did not observe differences in sex, age, education, income, BMI, physical activity, blood group and diabetes (Table 1).

**Table 1:** Characteristics of the sample population stratified by the use of psychotropic medications.

Characteristic	All sample (*n* = 791)	Use of psychotropic medications (*n* = 42)	No use of psychotropic medications (*n* = 749)	*p* value
Symptoms, number	3.7 (±2.8)	5.5 (±2.6)	3.6 (±2.8)	<0.001
Men	403 (51%)	23 (55%)	380 (55%)	0.727
Age, years	40.0 (±12.5)	43.0 (±11.7)	39.8 (±12.5)	0.105
Only primary school	407 (51%)	19 (45%)	388 (51%)	0.503
Lowest tertile income (<3,000€/month)	128 (16%)	6 (14%)	122 (16%)	0.898
Unemployed	163 (21%)	11 (26%)	152 (21%)	0.469
BMI, mean (SD), kg/m2	25.5 (±4.7)	26.2 (±4.6)	25.5 (±4.7)	0.316
Obesity	114 (14%)	6 (14%)	108 (14%)	1.000
Physical activity (MET-h/week)	14.5 (±10.0)	15.1 (±10.6)	14.5 (±10.0)	0.707
Lowest tertile of physical activity	248 (31%)	14 (33%)	234 (31%)	0.910
Current smoker	144 (18%)	13 (31%)	131 (18%)	0.016
Former smoker	147 (19%)	11 (26%)	136 (19%)	0.016
Never smoker	500 (63%)	18 (43%)	482 (63%)	0.016
Blood group A	286 (36%)	10 (24%)	276 (36%)	0.122
Diabetes	22 (3%)	3 (7%)	19 (3%)	0.199
Multimorbidity	72 (9%)	11 (26%)	61 (9%)	<0.001
Weight loss	99 (13%)	8 (19%)	91 (13%)	0.282
Polypharmacy	62 (8%)	8 (19%)	54 (8%)	0.013

MET, metabolic equivalent task. ^*^p value calculated with Chi squared test among classes for categorical variables and non-paired t test for continuous variables.

By comparing men and women, we found that men reported fewer baseline symptoms, were more frequently obese and inactive and were more regularly current smokers with diabetes and polypharmacy than women (Supplementary Table S2).

After testing the latent class model with four different link functions, we found the AIC were 30,958, 22,367, 24, 263 and 21,667 for linear distribution, beta distribution (concave, convex or sigmoïd transformations), spline distribution (5 equidistant knots) and spline with 3 equidistant nodes, respectively. We chose the model with the lowest AIC value, the spline with 3 knots at quantiles. Then, by fitting seven models with 3-equidistant spline models with trajectories from 1 to 7. Supplementary Table 3 details the result of the process of selection of classes. It shows the model with 3 knots fitted with 1 to 7 trajectories. The spline model with 3 knots and four symptom trajectories was chosen (lowest BIC and more than 5% in each class).

**Figure 1:**
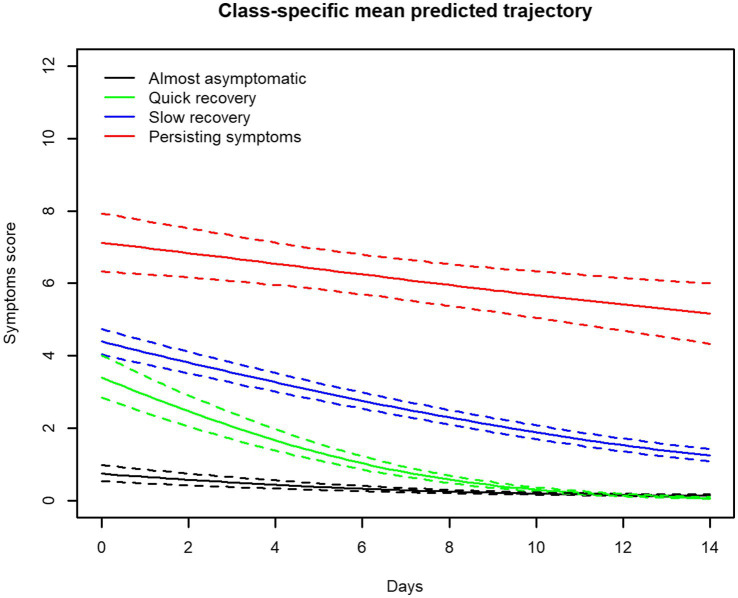
Symptom trajectories of 791 adults tested positive for SARS-CoV-2. The symptoms were reported (asked from a list of 16 symptoms) at baseline and every day after the baseline during 14 days.

Figure 1 shows the four symptom trajectories. “Almost asymptomatic” characterized people with very few baseline symptoms with a course of symptoms that did not increase or decrease. “Quick recovery” characterized people who seemed to recover remarkably well, with slight to moderate symptoms that tended to decline to achieve the same level as “Almost asymptomatic.” “Slow recovery” characterized people with mild to moderate baseline symptoms, with a tendency to recover but less quickly than that observed in the “Quick recovery symptom trajectory. “Persisting symptoms” characterized people who started with moderate or severe symptoms and failed to recover in 2 weeks, remaining at a high abnormal level of symptoms at the end of the follow-up.

Our model showed that the mean of the true positives was 0.81, with true positives ranging from 0.88 (“Persisting symptoms “trajectory given “Persisting symptoms“) to 0.72 (“Quick recovery” given “Quick recovery”). The mean of the false positives was 0.06 and ranged from 0.18: “Almost asymptomatic” given “Quick recovery” to 0: “Persisting symptoms “given “Almost asymptomatic” and “Persisting symptoms“given “Quick Recovery (Supplementary Figure S2).

The symptom score ranged from 0 to 13 points at baseline, and the median (IQR) was 3.5 (1.5, 5.5) points and varied according to the trajectory. Supplementary Figure S3 shows baseline symptoms for the total sample and by symptom trajectory. The most frequent symptoms at baseline in the total sample were muscle ache (44.6%), other symptoms (41.0%) and cough (36.8%). Baseline symptoms were more frequent in the “Persisting Symptoms” trajectory than in other trajectories. In particular, the pain was frequent in the “Persisting symptoms“trajectory (69.4%) and less frequent in the other trajectories (11.6, 24.2 and 33.5% in the “Almost asymptomatic,” “Quick recovery,” and “Slow recovery” trajectories, respectively. Also, fatigue was overrepresented in the “Persisting Symptoms” trajectory.

Table 2 shows the general characteristics of people in each trajectory. The “Almost asymptomatic” symptom trajectory (*n* = 264) showed the lowest frequency of PM (n = 4, 2%) and the lowest mean number of symptoms at baseline (1.0 (±1.3)). In this trajectory, men were more represented (*n* = 161, 66%), people had the lowest frequency of unintentional weight loss (*n* = 18, 7%), and were more frequently unemployed (*n* = 64 (26%).

**Table 2:** Characteristic s of the population by trajectory of symptoms.

Variable	All sample (*n* = 791)	Almost asymptomatic (*n* = 244)*	Quick recovery (*n* = 178)	Slow recovery (*n* = 315)	Persisting symptoms (*n* = 54)	*p* value
	*N* (%) or Mean (±SD)	*N* (%) or Mean (±SD)	*N* (%) or Mean (±SD)	*N* (%) or Mean (±SD)	*N* (%) or Mean (±SD)	
Psychotropic medications	42 (5%)	4 (2%)	7 (4%)	23 (7%)	8 (15%)	<0.001
Symptoms at baseline	3.7 (±2.8)	1.0 (±1.3)	4.3 (±2.0)	5.0 (±2.4)	6.8 (±3.0)	<0.001
Men	403 (51%)	161 (66%)	84 (47%)	140 (44%)	18 (33%)	<0.001
Age, mean (SD), years	40.0 (±12.5)	40.5 (±13.5)	37.3 (±11.3)	40.6 (±12.5)	43.5 (±10.3)	0.003
Only primary school	407 (51%)	127 (52%)	101 (57%)	157 (50%)	22 (41%)	0.184
Lowest tertile income (<3,000€/month)	128 (16%)	42 (17%)	28 (16%)	52 (17%)	6 (11%)	0.737
Unemployed	163 (21%)	64 (26%)	35 (20%)	57 (18%)	7 (13%)	0.047
BMI, mean (SD), kg/m^2^	25.5 (±4.7)	25.7 (±4.5)	25.3 (±4.4)	25.3 (±4.7)	26.8 (±6.0)	0.121
Obesity	114 (14%)	41 (17%)	25 (14%)	39 (12%)	9 (17%)	0.489
Physical activity, mean (SD) (MET-h/week)	14.5 (±10.0)	14.7 (±10.3)	14.1 (±9.7)	14.5 (±9.7)	14.9 (±10.9)	0.927
Lowest tertile of physical activity	248 (31%)	77 (32%)	58 (33%)	94 (30%)	19 (35%)	0.842
Current smoker	144 (18%)	51 (21%)	27 (15%)	59 (19%)	7 (13%)	0.551
Former smoker	147 (19%)	38 (16%)	38 (21%)	60 (19%)	11 (20%)	0.551
Never smoker	500 (63%)	155 (64%)	113 (63%)	113 (63)	11 (67%)	0.551
Blood group A	286 (36%)	99 (41%)	60 (34%)	107 (34%)	20 (37%)	0.362
Diabetes	22 (3%)	8 (3%)	2 (1%)	7 (2%)	5 (9%)	0.013
Multimorbidity	72 (9%)	21 (9%)	11 (6%)	30 (10%)	10 (19%)	0.051
Weight loss	99 (13%)	18 (7%)	18 (10%)	53 (17%)	10 (19%)	0.003
Polypharmacy	62 (8%)	20 (8%)	10 (6%)	24 (8%)	8 (15%)	0.178

MET, metabolic equivalent task.

“Quick recovery” trajectory (*n* = 178) was characterized by a low frequency of PM (n = 7, 4%) and a higher frequency of baseline symptoms (5.3 (±2.0)) than “Almost asymptomatic,” the lowest mean age (37.3 (±11.3)), the lowest frequencies of diabetes (*n* = 2, 1%), multimorbidity (n = 11, 6%) and polypharmacy (*n* = 10, 6%).

“Slow recovery” trajectory (*n* = 306) with similar baseline mean symptoms (5.0 (±2.4)) to “Quick recovery,” had higher frequencies of PM (*n* = 23, 7%), and weight loss (n = 53, 17%) compared to “Almost Asymptomatic.”

“Persisting symptoms” trajectory (*n* = 54) presented the highest mean number of symptoms at baseline among the other trajectories (6.8 (±3.0)), and compared to other trajectories had the highest frequencies of PM (*n* = 8, 15%), women (*n* = 36, 67%), diabetes (*n* = 5, 9%), multimorbidity (*n* = 10, 19%), weight loss (*n* = 10, 19%), and polypharmacy (*n* = 8, 15%). They also had the lowest frequencies of unemployment (*n* = 7, 13%).

We found that the most frequent psychotropic medication was Sertraline, and the most frequent type of psychotropic was antidepressants (Supplementary Table S4). The density distribution of the symptom score at baseline was different in the population with or without PM, with a median value of 3 and 5 for the population without and with PM, respectively. (Supplementary Figure S4). The percentage of people taking PM varied according to the trajectory of symptoms, being more frequent in the symptom trajectories “Persisting Symptoms” and “Slow Recovery” and, in particular, it was antidepressants and anxiolytics (Supplementary Figure S5).

Table 3 shows the association of PM with the risk of belonging to a symptom trajectory using a multivariate polynomial analysis. The dependent variable was the trajectory with the “Almost Asymptomatic” as the reference level, and the predictor was PM. With the most adjusted model, we found that PM was significantly associated with a higher risk of belonging to “Quick recovery” [RR 3.1 (95% CI 2.7, 3.4)], “Slow recovery” [RR 5.2 (95% CI 3.0, 9.2)] and “Persisting Symptoms” [RR 11.7 (95% CI 6.9, 19.6)] than “Almost asymptomatic” trajectory.

**Table 3:** Use of psychotropic medications to predict belonging to a trajectory: multivariate polynomial analysis.^*^

	Quick recovery (*n* = 178)	Slow recovery (*n* = 315)	Persisting symptoms (*n* = 54)
Models^a^	RR (95% CI)^b^	RR (95% CI) ^b^	RR (95% CI) ^b^
Model 0	2.5 (0.8, 7.2)	4.7 (3.5, 6.5)	10.4 (3.0, 35.9)
Model 1	2.8 (2.8, 2.9)	5.2 (2.8, 9.5)	11.6 (3.9, 34.1)
Model 2	3.1 (2.7, 3.4)	5.2 (3.0, 9.2)	11.7 (6.9, 19.6)

Abbreviations: RR = relative risk. 95% CI = 95% confidence intervals. ^a^Almost Asymptomatic was the reference level for trajectories. Model 0: Only main predictor; Model 1 = Model 0 adjusted by age and sex (not in stratified sex models); Model 2 = Model 1 adjusted by work status, income, education, smoking status, BMI, physical activity and multimorbidity. ^b^Confidence intervals were calculated according to Rubin’s rules.

## 4. Discussion

In this cohort study, PM was associated with more severe symptom trajectories. In people with PM, we observed poorer recovery during the first 2 weeks after the infection, even after adjusting for relevant confounders. We identified four trajectories of COVID-19 severity, with a score reflecting the reported total number of symptoms. We found that the symptom trajectories and recovery were heterogeneous and identified groups of people within these trajectories. This study is the first to perform latent class trajectory analysis of early COVID-19 symptoms.

Carrat et al. found among a group of risk factors that preexisting anxiety was associated with COVID-19-Like Symptoms (21). Castro et al., in a retrospective longitudinal analysis, found that people with preexisting mood disorders had a higher risk of COVID-19 mortality risk beyond day 12 after hospitalization (hazard ratio 1.540, 95% CI = 1.054, 2.250)) (22). Jeon et al. found that preexisting mental disorders were not associated with a higher susceptibility to COVID-19 infection but with mortality (23). Finally, Nishimi et al. studied 263,697 fully vaccinated patients and found that preexisting psychiatric disorders were associated with an increased incidence of COVID-19 infection (24). A systematic review and meta-analysis in COVID-19 found an association (cross-sectional or longitudinal) between preexisting mood or sleep disorders with a higher susceptibility to infection (pooled odds ratio 27 studies (95% confidence intervals) 1.67, (1.12, 2.49)), higher severity (21 studies 1.40 (1.25, 1.57)) and increased risk of death (29 studies 1.47 (1.26, 1.72)) (25). Our results agree with this previous research showing a more severe form of COVID-19 in people with increased psychological distress. These associations between anxiety or mood disorders and COVID-19 infection are likely bidirectional (26). Finally, a recent report analyzing 9,979 individuals diagnosed with COVID-19 showed that bedridden for more than 7 days had a higher risk of future depression than those who were never bedridden. These results highlight the bidirectional associations between depression and COVID-19 severity (27).

Previous reports on COVID-19 found particular trajectories of psychological disorders, identifying groups of individuals sharing a specific evolution over time (28, 29). A cross-sectional analysis including 938 health care workers found a higher prevalence of psychological disorders (stress, depression, and anxiety) in COVID-19 infected health workers (30).

A recent study found that psychological distress during the first wave of COVID-19 was associated with the belief of having had a COVID-19 infection, reporting a higher number and more severe symptoms attributed to COVID-19 (12). Taquet et al., using electronic health data, including 62,354 people with a COVID-19 diagnosis, found that psychological disturbances had bidirectional associations with COVID-19 infection. There was an association between a pre-existing psychiatric condition and incident COVID-19 [relative risk = 1.5 (95% CI 1.5–1.71)] (26). Another study also used health records and found a similar association between pre-existing psychological disorders and higher risk for COVID-19 infection, hospitalization and mortality. The most substantial effect was observed in depression and future infection risk. They found an adjusted odds ratio of 7.64, 95% CI: 7.45–7.83, *p* < 0.001) (14). With data from the UK Biobank, Wang et al. found an association of preexisting mental disorders and COVID-19 incidence and severity. Anxiety [OR 1.29 (95% CI 1.17–1.42)] and depression [OR 1.22 (95% CI 1.13–1.31)] were associated with a higher risk of infection. Depression was also associated with a higher mortality risk post COVID-19 [OR 1.57 (95% CI 1.16–2.13)] (31).

An observational study on adults with psychiatric diagnoses and severe COVID-19 disease found that taking functional inhibitors of acid sphingomyelinase, a type of psychotropic, was associated with lower mortality in severe COVID-19 cases (32). We could not analyze whether there were differences according to the psychotropic medications due to the small number of people taking them in our sample.

Our findings agree with these studies, and we found, in addition, an association between PM and symptom trajectories. Among psychotropic medications, we observed that antidepressant and anxiolytics were overrepresented in the most severe symptom trajectories. Our results suggest that the symptoms trajectory would vary depending on the type of psychiatric diagnosis, observing more pronounced differences with more depression or anxiety diagnoses than psychotic or neurologic disorders. We think that the relationship between psychological disturbance and COVID-19 is bidirectional. The effect of PM on the symptom trajectories was higher in magnitude in “Persisting symptoms,” suggesting that mood disorders/anxiety are associated with greater disease severity and poorer recovery.

We observed that pain and fatigue were over-represented in the “Persisting symptoms” trajectory. This finding aligns with two previous research that has shown associations between depressive symptoms and post-COVID-19 fatigue (33) and pain symptoms (34) 3 months and 1 year after COVID-19 infection, respectively. Our findings show that the early evolution of COVID-19 can help predict later evolution.

We found that the overall tendency was to reduce symptoms, but with different recovery curves, some people achieved an almost total recovery while others achieved only partial recovery. Our findings align with a study that characterized trajectories of symptoms in the first weeks post-infection from SARS-CoV-2. They found similar results, although their analysis was about individual symptom trajectories and not a total score to assess overall disease severity (2). The healthiest group corresponds to “Quick recovery.” We also found that unemployed people were mainly in the less risky “Almost asymptomatic” trajectory. These findings can be explained because unemployed people were less exposed to the virus.

We identified four distinct symptom trajectories. Previous research in a population of COVID-19 patients, 94% hospitalized and a mean age of 64 years, showed that men were more at risk of developing severe COVID-19 disease. They also observed that testosterone levels in men were inversely associated with severity (35). We found that “Almost asymptomatic” were over-represented by men. A possible explanation is that our population was much younger (mean age 39 years) and primarily asymptomatic or with mild disease (77%) compared to the previous study.

The underlying mechanisms that could explain the association between depression and COVID-19 severity are that they share inflammatory pathways with an increase in inflammatory biomarkers such as TNFα, interleukin 1-β and interleukin-6 (36). A possible mechanism for explaining the association between pre-existing anxiety and COVID-19 severity could be a higher neutrophil/lymphocyte ratio in patients with anxiety. Through a cortisol elevation, psychosocial stress is also associated with decreased immunity and a decrease in some cytokines (37). In animal models, the associated anxiety-induced reduction of immunity is not restricted to the cellular but also the humoral response (38).

Finally, there is evidence of the association of olfactory function through nasal inflammation and neuropsychiatric diagnoses, which is relevant because of the olfactory compromise of COVID-19 and perhaps due to vaccination (39, 40).

### 4.1. Strengths and limitations

This study has several strengths. In this cohort study, the questionnaires were online, and the COVID-19 diagnosis was ascertained with a PCR test. We analyzed a large sample size representing a large set of COVID-19-related symptoms tracked during 14 days with an innovative methodology to simultaneously characterize disease severity and recovery.

This study also has several limitations. The symptoms, determinants and confounders were self-reported, which could introduce reporting bias. Only 5% of the population reported PM, which limited the analysis to the total sample, making it impossible for relevant stratified analysis. The observed associations are strong in magnitude, but these results would now require confirmation in other populations, where this type of medication is frequent. We observed missing data and loss of follow-up, which might introduce biases. However, we performed multiple imputations with a state-of-the-art method. Multiple imputation techniques help reduce the bias if a complete-case analysis is performed. They also help increase the power of the analysis because each time there is an unanswered question in a questionnaire, there is a loss of information for calculating the total score (41). Our population did not include much older people, and few participants were recruited at the hospital, making it difficult to directly compare with previous works performed in hospital-based cohort studies with more severe cases. However, this study provides significant findings for the general population. Finally, with the unpredictable evolution of the pandemic due to vaccine discovery, the virus mutations and the surveillance or lack of it, likely, our findings may not fully represent what is going to be the future disease trajectories (42).

### 4.2. Conclusion

This study described four distinct symptom trajectories of COVID-19 with different recovery timing. We also showed that PM before the infection was associated with a greater risk of disease severity and a poorer recovery in the first 2 weeks. In addition to all the established risk factors of COVID-19, our findings could help identify at-risk individuals and personalize prevention strategies and care in case of infection to SARS-COV-2. The results of our study can be generalizable to a similar adult population of European origin.

## Data availability statement

The raw data supporting the conclusions of this article will be made available by the authors, without undue reservation.

## Ethics statement

The studies involving human participants were reviewed and approved by The National Research Ethics Committee of Luxembourg (CNER). The patients/participants provided their written informed consent to participate in this study.

## Author contributions

GA contributed to the data curation, formal analysis, methodology, visualization, and writing the original draft. AF contributed to the conceptualization, data curation, and review and editing. AE contributed to data curation and review and editing. NL contributed to writing the original draft and reviewing and editing. MO contributed to conceptualization and review and editing. GF contributed to conceptualization, funding acquisition, methodology, supervision, and review and editing. All authors contributed to the article and approved the submitted version.

## Funding

The Predi-COVID study is supported by the Luxembourg National Research Fund (FNR) (Predi-COVID, grant number 14716273), the André Losch Foundation and by European Regional Development Fund (FEDER, convention 2018-04-026-21). The work was further supported by the Luxembourg Government through the CoVaLux programme.

## Conflict of interest

The authors declare that the research was conducted in the absence of any commercial or financial relationships that could be construed as a potential conflict of interest.

## Publisher’s note

All claims expressed in this article are solely those of the authors and do not necessarily represent those of their affiliated organizations, or those of the publisher, the editors and the reviewers. Any product that may be evaluated in this article, or claim that may be made by its manufacturer, is not guaranteed or endorsed by the publisher.

## References

[1] Del Rio C Collins LF Malani P. Long-term health consequences of COVID-19. JAMA. (2020) 324:1723–4. doi: 10.1001/jama.2020.19719, PMID: 33031513 PMC8019677

[2] Rodebaugh TL Frumkin MR Reiersen AM Lenze EJ Avidan MS Miller JP. Acute Symptoms of Mild to Moderate COVID-19 are Highly Heterogeneous Across Individuals and Over Time. Open Forum Infectious Diseases. United States: Oxford University Press (2021). 10.1093/ofid/ofab090 PMC7989225 33796601

[3] Sudre CH Lee KA Lochlainn MN Varsavsky T Murray B Graham MS. Symptom clusters in COVID-19: a potential clinical prediction tool from the COVID symptom study app. Sci Adv. (2021) 7:eabd4177. doi: 10.1126/sciadv.abd4177, PMID: 33741586 PMC7978420

[4] Cohen K Ren S Heath K Dasmariñas MC Jubilo KG Guo Y. Risk of persistent and new clinical sequelae among adults aged 65 years and older during the post-acute phase of sars-cov-2 infection: retrospective cohort study. BMJ. (2022); 376. e068414. doi: 10.1136/bmj-2021-068414 PMC8828141 35140117

[5] Guo P Benito Ballesteros A Yeung SP Liu R Saha A Curtis L. COVCOG 1: factors predicting physical, neurological and cognitive symptoms in long COVID in a community sample. A first publication from the COVID and cognition study. Front Aging Neurosci. (2022) 14:14. doi: 10.3389/fnagi.2022.804922 PMC8968323 35370617

[6] Sudre CH Murray B Varsavsky T Graham MS Penfold RS Bowyer RC. Attributes and predictors of long COVID. Nat Med. (2021) 27:626–31. doi: 10.1038/s41591-021-01292-y, PMID: 33692530 PMC7611399

[7] Galal I Hussein AAM Amin MT Saad MM Zayan HEE Abdelsayed MZ. Determinants of persistent post-COVID-19 symptoms: value of a novel COVID-19 symptom score. Egypt J Bronchol. (2021) 15:1–8. doi: 10.1186/s43168-020-00049-4

[8] Cuomo A Amore M Arezzo MF De Filippis S De Rose A La Pia S. Mental health in Italy after two years of COVID-19 from the perspective of 1281 Italian physicians: looking back to plan forward. Ann General Psychiatry. (2022) 21:30. doi: 10.1186/s12991-022-00410-5, PMID: 35948983 PMC9363263

[9] Fagiolini A Cuomo A Frank E. COVID-19 diary from a psychiatry department in Italy. J Clin Psychiatry. (2020) 81:3909. doi: 10.4088/JCP.20com13357 32237301

[10] Kim J-W Kang H-J Jhon M Ryu S Lee J-Y Kang S-J. Associations between CoViD-19 symptoms and psychological distress. Front Psych. (2021) 12:1401. doi: 10.3389/fpsyt.2021.721532 PMC8415963 34484008

[11] Thye AY-K Law JW-F Tan LT-H Pusparajah P Ser H-L Thurairajasingam S. Psychological symptoms in COVID-19 patients: insights into pathophysiology and risk factors of long COVID-19. Biology. (2022) 11:61. doi: 10.3390/biology11010061, PMID: 35053059 PMC8773222

[12] Ayling K Jia R Coupland C Chalder T Massey A Broadbent E. Psychological predictors of self-reported COVID-19 outcomes: results from a prospective cohort study. Ann Behav Med. (2022) 56:484–97. doi: 10.1093/abm/kaab106, PMID: 34979556 PMC8755370

[13] Cañas-González B Fernández-Nistal A Ramírez JM Martínez-Fernández V. Influence of stress and depression on the immune system in patients evaluated in an anti-aging unit. Front Psychol. (1844) 11:1844. doi: 10.3389/fpsyg.2020.01844 PMC7417678 32849086

[14] Wang Q Xu R Volkow ND. Increased risk of COVID-19 infection and mortality in people with mental disorders: analysis from electronic health records in the United States. World Psychiatry. (2021) 20:124–30. doi: 10.1002/wps.20806, PMID: 33026219 PMC7675495

[15] Fagherazzi G Fischer A Betsou F Vaillant M Ernens I Masi S. Protocol for a prospective, longitudinal cohort of people with COVID-19 and their household members to study factors associated with disease severity: the Predi-COVID study. BMJ Open. (2020) 10:e041834. doi: 10.1136/bmjopen-2020-041834, PMID: 33234656 PMC7684799

[16] Buuren S Groothuis-Oudshoorn K. MICE: multivariate imputation by chained equations in R. J Stat Softw. (2011) 45:1–67. doi: 10.18637/jss.v045.i03

[17] Van Buuren S Brand JPL Groothuis-Oudshoorn CGM Rubin DB. Fully conditional specification in multivariate imputation. J Stat Comput Simul. (2006) 76:1049–64. doi: 10.1080/10629360600810434

[18] Jung T Wickrama KA. An introduction to latent class growth analysis and growth mixture modeling. Soc Personal Psychol Compass. (2008) 2:302–17. doi: 10.1111/j.1751-9004.2007.00054.x

[19] Weller BE Bowen NK Faubert SJ. Latent class analysis: a guide to best practice. J Black Psychol. (2020) 46:287–311. doi: 10.1177/0095798420930932

[20] Von Elm E Altman DG Egger M Pocock SJ Gøtzsche PC Vandenbroucke JP. The strengthening the reporting of observational studies in epidemiology (STROBE) statement: guidelines for reporting observational studies. Int J Surg. (2014) 12:1495–9. doi: 10.1016/j.ijsu.2014.07.013 25046131

[21] Carrat F Touvier M Severi G Meyer L Jusot F Lapidus N. Incidence and risk factors of COVID-19-like symptoms in the French general population during the lockdown period: a multi-cohort study. BMC Infect Dis. (2021) 21:1–13. doi: 10.1186/s12879-021-05864-8 33568097 PMC7875161

[22] Castro VM Gunning FM McCoy TH Perlis RH. Mood disorders and outcomes of COVID-19 hospitalizations. Am J Psychiatr. (2021) 178:541–7. doi: 10.1176/appi.ajp.2020.20060842 33820425 PMC11905964

[23] Jeon H-L Kwon JS Park S-H Shin J-Y. Association of mental disorders with SARS-CoV-2 infection and severe health outcomes: nationwide cohort study. Br J Psychiatry. (2021) 218:344–51. doi: 10.1192/bjp.2020.251, PMID: 33407954

[24] Nishimi K Neylan TC Bertenthal D Seal KH O’Donovan A. Association of Psychiatric Disorders with Incidence of SARS-CoV-2 breakthrough infection among vaccinated adults. JAMA Netw Open. (2022) 5:e227287-e. doi: 10.1001/jamanetworkopen.2022.7287, PMID: 35420660 PMC9011123

[25] Liu L Ni S-Y Yan W Lu Q-D Zhao Y-M Xu Y-Y. Mental and neurological disorders and risk of COVID-19 susceptibility, illness severity and mortality: a systematic review, meta-analysis and call for action. EClinicalMedicine. (2021) 40:101111. doi: 10.1016/j.eclinm.2021.101111, PMID: 34514362 PMC8424080

[26] Taquet M Luciano S Geddes JR Harrison PJ. Bidirectional associations between COVID-19 and psychiatric disorder: retrospective cohort studies of 62 354 COVID-19 cases in the USA. Lancet Psychiatry. (2021) 8:130–40. doi: 10.1016/S2215-0366(20)30462-4, PMID: 33181098 PMC7820108

[27] Magnúsdóttir I Lovik A Unnarsdóttir AB McCartney D Ask H Kõiv K. Acute COVID-19 severity and mental health morbidity trajectories in patient populations of six nations: an observational study. The lancet. Public Health. (2022) 7:e406–16. doi: 10.1016/S2468-2667(22)00042-1 35298894 PMC8920517

[28] Pierce M McManus S Hope H Hotopf M Ford T Hatch SL. Mental health responses to the COVID-19 pandemic: a latent class trajectory analysis using longitudinal UK data. Lancet Psychiatry. (2021) 8:610–9. doi: 10.1016/S2215-0366(21)00151-6, PMID: 33965057 PMC9764381

[29] Fernández-de-Las-Peñas C Martín-Guerrero JD Cancela-Cilleruelo I Moro-López-Menchero P Rodríguez-Jiménez J Pellicer-Valero OJ. Trajectory curves of post-COVID anxiety/depressive symptoms and sleep quality in previously hospitalized COVID-19 survivors: the LONG-COVID-EXP-CM multicenter study. Psychol Med (2022): 1–2. doi: 10.1017/S003329172200006X 10.1017/S003329172200006X PMC8770842 35000650

[30] Mohammadian Khonsari N Shafiee G Zandifar A Mohammad Poornami S Ejtahed H-S Asayesh H. Comparison of psychological symptoms between infected and non-infected COVID-19 health care workers. BMC Psychiatry. (2021) 21:1–9. doi: 10.1186/s12888-021-03173-7 33771122 PMC7995388

[31] Wang Y Yang Y Ren L Shao Y Tao W Dai X-J. Preexisting mental disorders increase the risk of COVID-19 infection and associated mortality. Front Public Health. (2021) 9:1134. doi: 10.3389/fpubh.2021.684112 PMC8381336 34434913

[32] Hoertel N Sánchez-Rico M Gulbins E Kornhuber J Carpinteiro A Abellán M. Association between FIASMA psychotropic medications and reduced risk of intubation or death in individuals with psychiatric disorders hospitalized for severe COVID-19: an observational multicenter study. Transl Psychiatry. (2022) 12:90. doi: 10.1038/s41398-022-01804-5, PMID: 35241663 PMC8892828

[33] Mazza MG Palladini M Villa G De Lorenzo R Querini PR Benedetti F. Prevalence, trajectory over time, and risk factor of post-COVID-19 fatigue. J Psychiatr Res. (2022) 155:112–9. doi: 10.1016/j.jpsychires.2022.08.008, PMID: 36029623 PMC9391361

[34] Bottemanne H Gouraud C Hulot J-S Blanchard A Ranque B Lahlou-Laforêt K. Do anxiety and depression predict persistent physical symptoms after a severe COVID-19 episode? A prospective study. Front Psych. (2021) 12:1875. doi: 10.3389/fpsyt.2021.757685 PMC8631493 34858230

[35] Dhindsa S Zhang N McPhaul MJ Wu Z Ghoshal AK Erlich EC. Association of circulating sex hormones with inflammation and disease severity in patients with COVID-19. JAMA Netw Open. (2021) 4:e2111398-e. doi: 10.1001/jamanetworkopen.2021.11398, PMID: 34032853 PMC8150664

[36] Mazza MG Palladini M Poletti S Benedetti F. Post-COVID-19 depressive symptoms: epidemiology, pathophysiology, and pharmacological treatment. CNS Drugs. (2022) 36:681–702. doi: 10.1007/s40263-022-00931-3 35727534 PMC9210800

[37] Nami M Mehrabi S Kamali A-M Kazemiha M Carvalho J Derman S. A new hypothesis on anxiety, sleep insufficiency, and viral infections; reciprocal links to consider in today's “world vs. COVID-19” endeavors. Front Psych. (2020) 11:585893. doi: 10.3389/fpsyt.2020.585893 PMC7674554 33250794

[38] Rammal H Bouayed J Falla J Boujedaini N Soulimani R. The impact of high anxiety level on cellular and humoral immunity in mice. Neuroimmunomodulation. (2010) 17:1–8. doi: 10.1159/000243079, PMID: 19816051

[39] Hasegawa Y Ma M Sawa A Lane AP Kamiya A. Olfactory impairment in psychiatric disorders: does nasal inflammation impact disease psychophysiology? Transl Psychiatry. (2022) 12:314. doi: 10.1038/s41398-022-02081-y, PMID: 35927242 PMC9352903

[40] Lechien JR Diallo AO Dachy B Le Bon SD Maniaci A Vaira LA. COVID-19: post-vaccine smell and taste disorders: report of 6 cases. Ear Nose Throat J. (2021):01455613211033125. doi: 10.1177/01455613211033125 34467793

[41] Perkins NJ Cole SR Harel O Tchetgen Tchetgen EJ Sun B Mitchell EM. Principled approaches to missing data in epidemiologic studies. Am J Epidemiol. (2018) 187:568–75. doi: 10.1093/aje/kwx348, PMID: 29165572 PMC5860376

[42] Telenti A Arvin A Corey L Corti D Diamond MS García-Sastre A. After the pandemic: perspectives on the future trajectory of COVID-19. Nature. (2021) 596:495–504. doi: 10.1038/s41586-021-03792-w, PMID: 34237771

