# Supplementary material for: Association between use of psychotropic medications prior to SARS-COV-2 infection and trajectories of COVID-19 recovery: Findings from the prospective Predi-COVID cohort study
Source: Front Public Health. 2023 Mar 16;11:1055440. doi: 10.3389/fpubh.2023.1055440 (PMC10062525; doi:10.3389/fpubh.2023.1055440)
Supplement: Supplementary file 1 [file Table_1.DOCX]

Supplementary Material

[1. Supplementary Figures 2](#_Toc126832667)

[Supplementary Figure 1. Flow diagram of included participants 2](#_Toc126832668)

[Supplementary Figure 2. Posterior classification trajectories 3](#_Toc126832669)

[Supplementary Figure 3. Baseline symptoms in each symptom trajectory 4](#_Toc126832670)

[Supplementary Figure 4. Density distribution of Covid-19 symptom score by psychotropic medication use 5](#_Toc126832671)

[Supplementary Figure 5. Frequency of psychotropic medication use by Covid-19 symptom trajectories 6](#_Toc126832672)

[Supplementary Table 1. Covid-19 symptoms daily questionnaire 7](#_Toc126832673)

[Supplementary Table 2. Total population stratified by sex 9](#_Toc126832674)

[Supplementary Table 3. Selection of trajectories 10](#_Toc126832675)

[Supplementary Table 4. Classification and frequency of use of psychotropic medications in Predicovid study 11](#_Toc126832676)

[STROBE Statement—checklist of items that should be included in reports of observational studies 12](#_Toc126832677)

# Supplementary Figures

## Supplementary Figure 1. Flow diagram of included participants


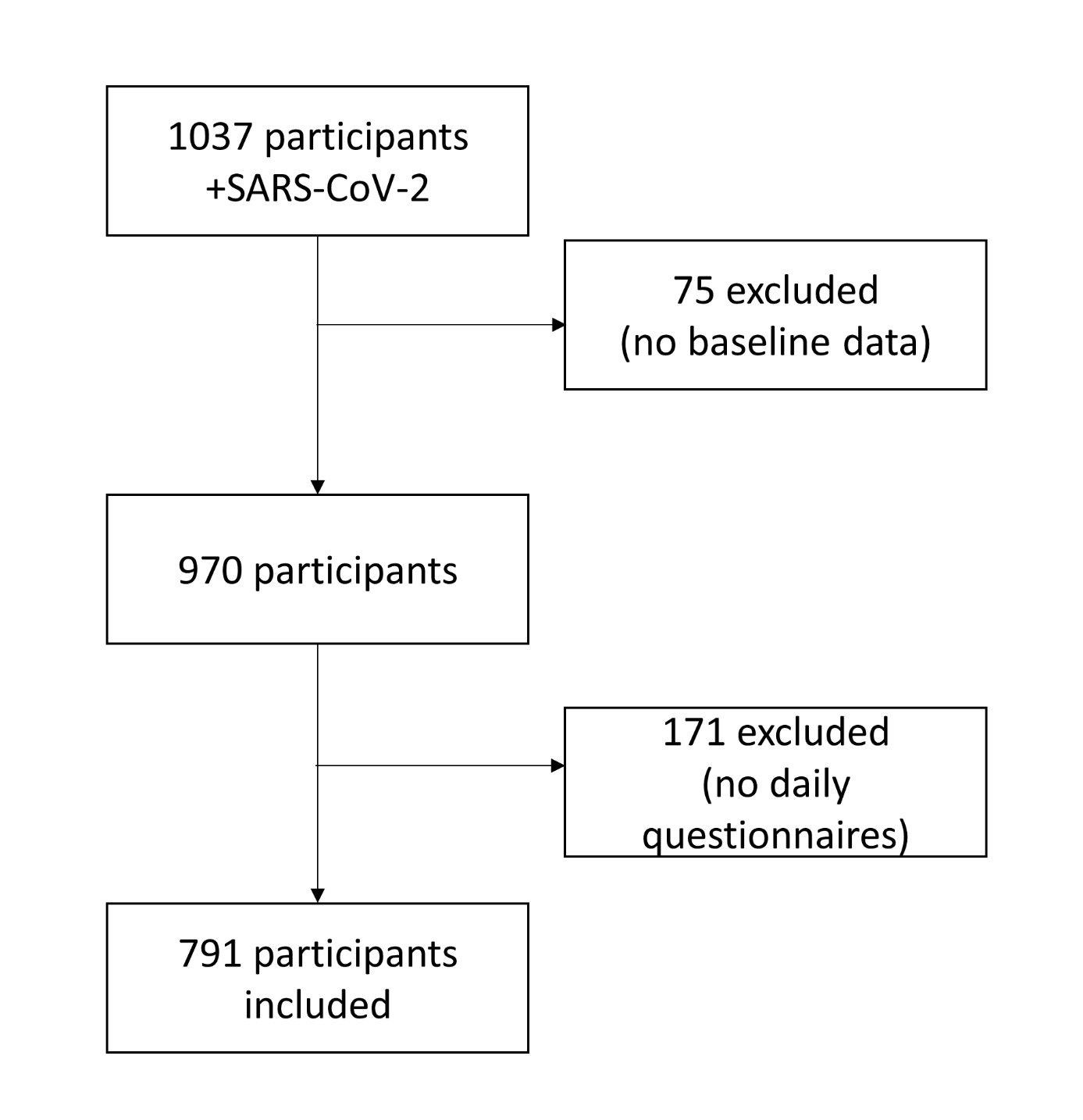


## Supplementary Figure 2. Posterior classification trajectories


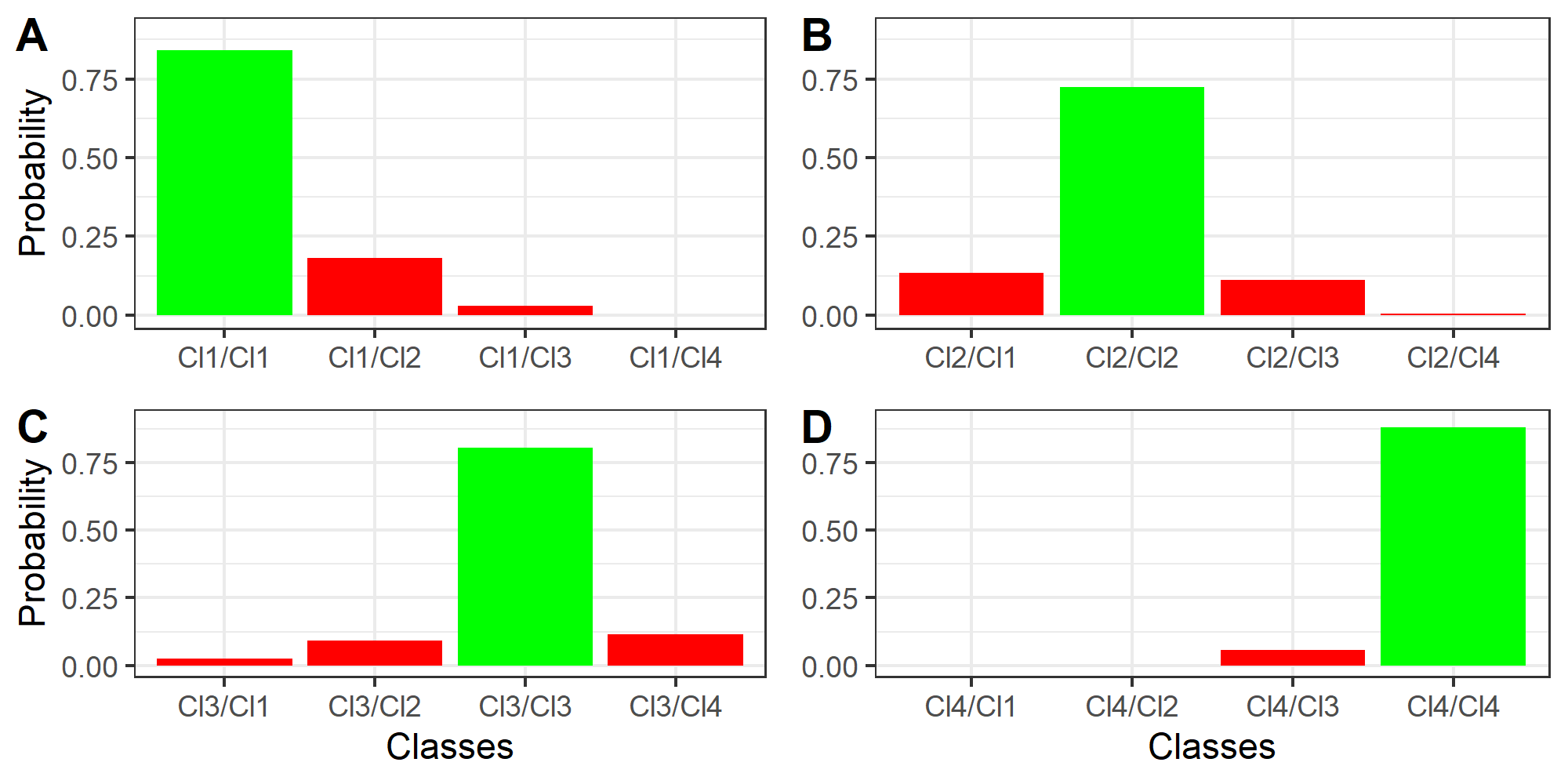


Abbreviations: Cl1: class 1 (Almost asymptomatic); Cl2: class 2 (Quick recovery); Cl3: class 3 (Slow recovery); Cl4: Class 4 (Persisting symptoms).

Panels A to D show posterior probabilities of belong to a class given the initial classification. Green and red bars show true and false positives, respectively.

##
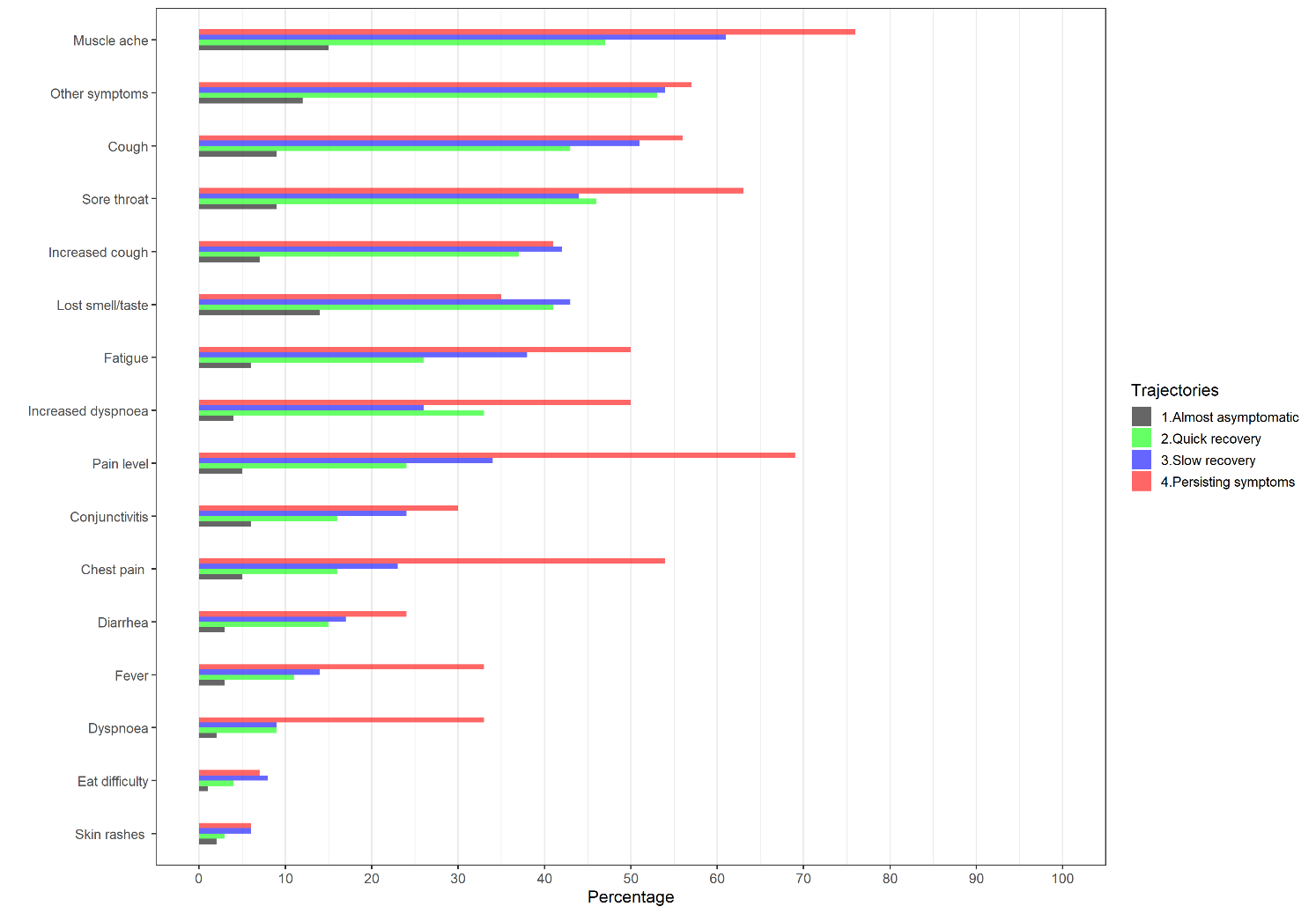
Supplementary Figure 3. Baseline symptoms in each symptom trajectory

The most common symptom at baseline was muscle aches. Participants that followed the “Persisting symptoms” (blue bars) trajectory showed more frequent baseline symptoms.

## Supplementary Figure 4. Density distribution of Covid-19 symptom score by psychotropic medication use


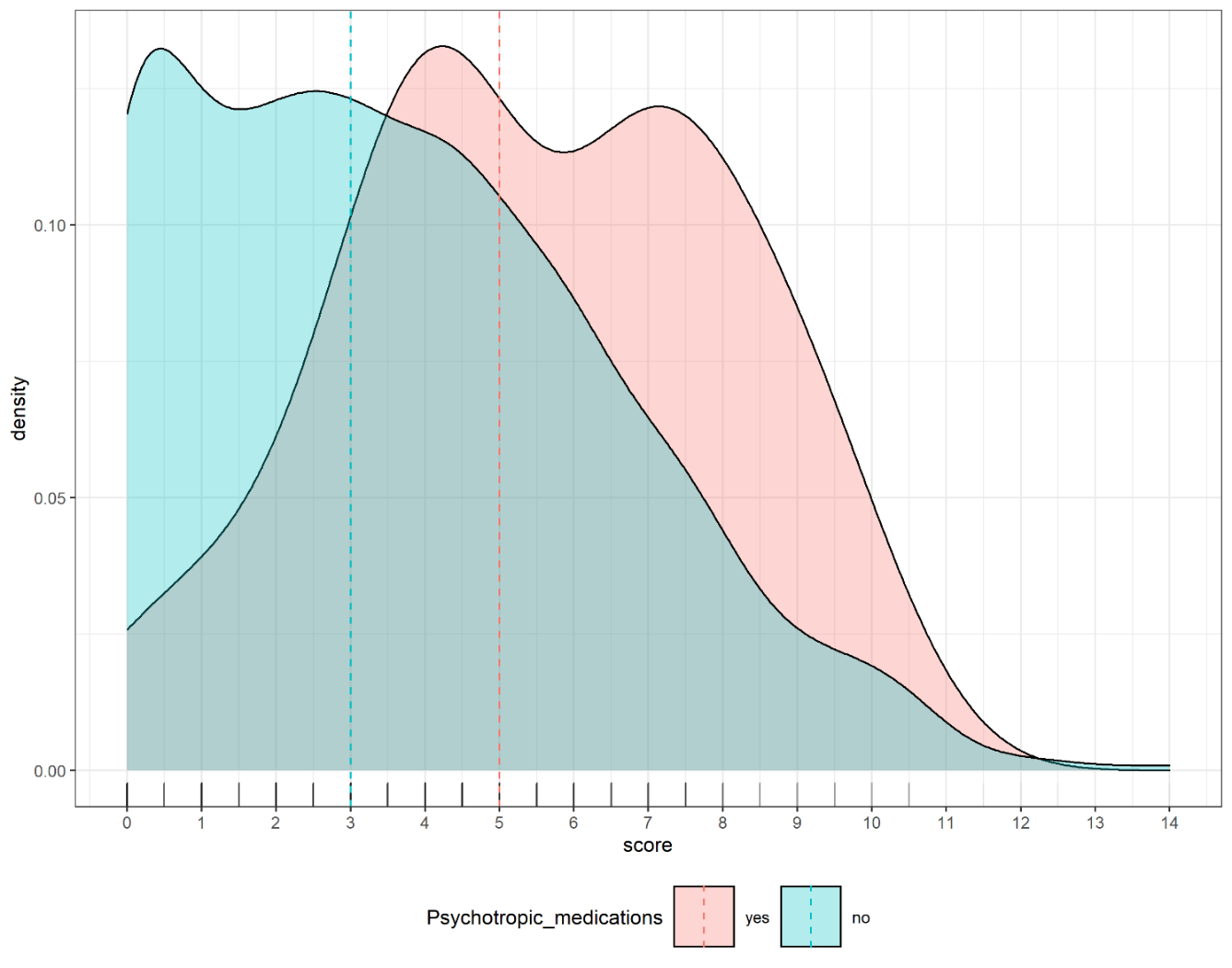


The density distribution of the symptom score at baseline of the participants that took psychotropic medications (in light red) was different compared to the density distribution of participants that did not take them (in blue).

## Supplementary Figure 5. Frequency of psychotropic medication use by Covid-19 symptom trajectories


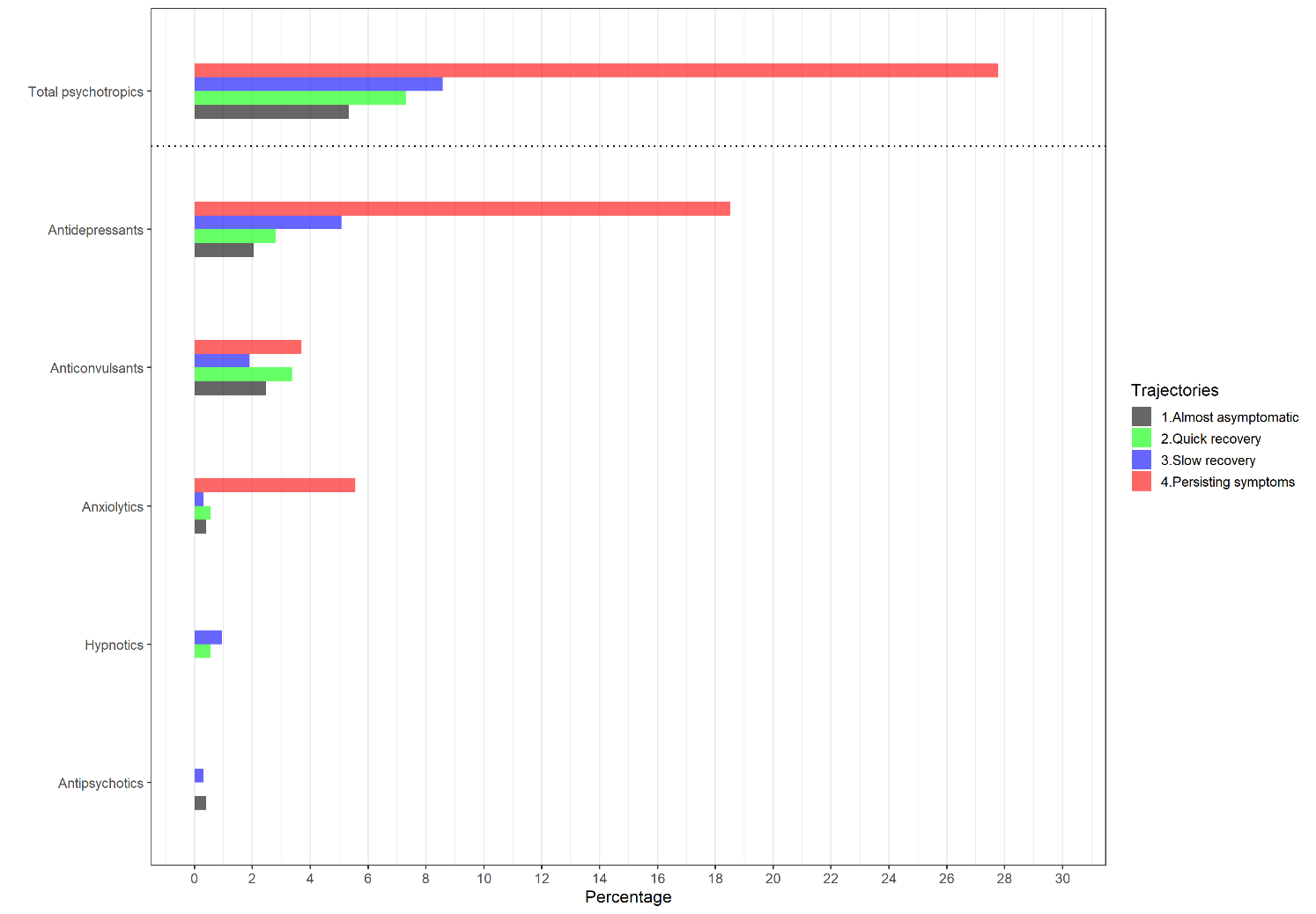


The most reported psychotropic medications were antidepressants. Participants that followed the “Persisting symptoms” trajectory reported more frequent use of antidepressants and anxiolytics compared with the other symptom trajectories.

**Supplementary Tables**

## Supplementary Table 1. Covid-19 symptoms daily questionnaire

| **Number** | **Question** | **Answer** | **Points** |
| --- | --- | --- | --- |
| 1 | How do you feel today? | I feel good | 0 |
|  |  | I feel tired | 0.5 |
|  |  | I feel bad | 1 |
| 2 | Do you have a dry cough? | Yes | 1 |
|  |  | No | 0 |
| 3 | Have you had an increase in your usual cough in recent days? | Yes | 1 |
|  |  | No | 0 |
| 4 | Have you had a sore throat in recent days? | Yes | 1 |
|  |  | No | 0 |
| 5 | Have you noticed a sharp decrease or loss of your taste or sense of smell? | Yes | 1 |
|  |  | No | 0 |
| 6 | Do you have diarrhea? With at least 3 liquid/soft stools per day? | Yes | 1 |
|  |  | No | 0 |
| 7 | Have you had unusual muscle aches or body aches in recent days? | Yes | 1 |
|  |  | No | 0 |
| 8 | Have you had chest pains lately? | Yes | 1 |
|  |  | No | 0 |
| 9 | What is your current pain level? Scale from 0 (no pain) to 10 ( maximum pain) | <2 | 0 |
|  |  | >=2 & <3 | 0.5 |
|  |  | >=3 | 1 |
| 10 | Do you have fever? | Yes | 1 |
|  |  | No | 0 |
| 11 | Do you have breathing difficulties? | Yes | 1 |
|  |  | No | 0 |
| 12 | Have you noticed the appearance of difficulty breathing or an increase in your usual difficulty breathing? | Yes | 1 |
|  |  | No | 0 |
| 13 | Do you have significant difficulty eating or drinking? | Yes | 1 |
|  |  | No | 0 |
| 14 | Have you noticed a sudden appearance of rashes on the hands or feet (e.g. chilblains, persistent redness sometimes painful, transient hives)? | Yes | 1 |
|  |  | No | 0 |
| 15 | Have you noticed the appearance of conjunctivitis or pain in  the eyes (persistent redness in the white of the eye, itching in the eyelids, tingling sensations, burning, and frequent tearing)? | Yes | 1 |
|  |  | No | 0 |
| 16 | Do you have any other symptoms? | Yes | 1 |
|  |  | No | 0 |

##

## Supplementary Table 2. Total population stratified by sex

| Characteristic | All sample (n=791) | Males (n=403) | Females (n=388) | P value |
| --- | --- | --- | --- | --- |
| Symptoms, number | 3.7 (±2.8) | 3.1 (±2.6) | 4.3 (±2.9) | >0.01 |
| Psychotropic medications | 42 (5%) | 23 (6%) | 19 (5%) | 0.727 |
| Age, years | 40 (±12.5) | 41 (±12.4) | 39 (±12.6) | 0.105 |
| Only primary school | 407 (51%) | 213 (53%) | 194 (50%) | 0.464 |
| Lowest tertile income (<3000€/month) | 128 (16%) | 55 (14%) | 73 (19%) | 0.061 |
| Unemployed | 163 (21%) | 83 (21%) | 80 (21%) | 1.000 |
| BMI, mean (SD), kg/m2 | 25.5 (±4.7) | 26.5 (±4.4) | 24.5 (±4.8) | 0.316 |
| Obesity | 114 (14%) | 73 (18%) | 41 (11%) | 0.003 |
| Physical activity (MET-h/week) | 14.5 (±10.0) | 13.9 (±9.8) | 15.2 (±11.0) | 0.707 |
| Lowest tertile of physical activity | 248 (31%) | 149 (37%) | 99 (26%) | 0.001 |
| Current smoker | 144 (18%) | 77 (19%) | 67 (17%) | >0.01 |
| Former smoker | 147 (19%) | 100 (25%) | 47 (12%) | >0.01 |
| Never smoker | 500 (63%) | 226 (56%) | 274 (71%) | >0.01 |
| Blood group A | 286 (36%) | 140 (35%) | 146 (38%) | 0.440 |
| Diabetes | 22 (3%) | 17 (4%) | 5 (1%) | 0.022 |
| Multimorbidity | 72 (9%) | 40 (10%) | 32 (8%) | 0.486 |
| Weight loss | 99 (13%) | 47 (12%) | 52 (13%) | 0.528 |
| Polypharmacy | 62 (8%) | 41 (105) | 21 (5%) | 0.018 |
| Abbreviations: UPM =Use of psychotropic medications. MET = metabolic equivalent task. *P value calculated with Chi squared test among classes for categorical variables and non-paired t test for continuous variables. | | | | |

## Supplementary Table 3. Selection of trajectories

| Trajectories | AIC | BIC | entropy | % class 1 | % class 2 | % class 3 | % class 4 | % class 5 | % class 6 | % class 7 |
| --- | --- | --- | --- | --- | --- | --- | --- | --- | --- | --- |
| 1 | 21667 | 21709 | 1.00 | 100 | NA | NA | NA | NA | NA | NA |
| 2 | 21621 | 21677 | 0.60 | 28 | 72 | NA | NA | NA | NA | NA |
| 3 | 21596 | 21666 | 0.52 | 34 | 33 | 33 | NA | NA | NA | NA |
| 4* | 21501 | 21585 | 0.67 | 23 | 7 | 40 | 31 | NA | NA | NA |
| 5 | 21507 | 21605 | 0.59 | 27 | 0 | 40 | 7 | 27 | NA | NA |
| 6 | 21697 | 21809 | 0.00 | 36 | 18 | 3 | 0 | 43 | 1 | NA |
| 7 | 21557 | 21683 | 0.51 | 0 | 2 | 28 | 36 | 0 | 25 | 9 |

*This was the final model

## Supplementary Table 4. Classification and frequency of use of psychotropic medications in Predicovid study

| **Medication** | **ATC code*** | **Use** | **%** |
| --- | --- | --- | --- |
| Sertraline | N06AB06 | Antidepressants | 21.4 |
| Escitalopram | N06AB10 | Antidepressants | 16.7 |
| Paroxetine | N06AB05 | Antidepressants | 11.9 |
| Venlafaxine | N06AX16 | Antidepressants | 9.5 |
| Mirtazapine | N06AX11 | Antidepressants | 7.1 |
| Fluoxetine | N06AB03 | Antidepressants | 2.4 |
| Trazodone | N06AX05 | Antidepressants | 2.4 |
| Bupropion | N06AX12 | Antidepressants | 2.4 |
| Duloxetine | N06AX21 | Antidepressants | 2.4 |
| Vortioxetine | N06AX26 | Antidepressants | 2.4 |
| Topiramate | N03AX11 | Anticonvulsants | 7.1 |
| Pregabalin | N03AX16 | Anticonvulsants | 4.8 |
| Lacosamide | N03AX18 | Anticonvulsants | 4.8 |
| Phenobarbital | N03AA02 | Anticonvulsants | 2.4 |
| Carbamazepine | N03AF01 | Anticonvulsants | 2.4 |
| Perampanel | N03AX22 | Anticonvulsants | 2.4 |
| Alprazolam | N05BA12 | Anxiolytics | 9.5 |
| Lorazepam | N05BA06 | Anxiolytics | 7.1 |
| Nordazepam | N05BA16 | Anxiolytics | 4.8 |
| Diazepam | N05BA01 | Anxiolytics | 2.4 |
| Zolpidem | N05CF02 | Hypnotics | 7.1 |
| Melatonin | N05CH01 | Hypnotics | 2.4 |
| Quetiapine | N05AH04 | Antipsychotics | 2.4 |
| Sulpiride | N05AL01 | Antipsychotics | 2.4 |
| *ATC code: Anatomical Therapeutic Chemical code | | |  |

## STROBE Statement—checklist of items that should be included in reports of observational studies

|  | Item No | Recommendation | Page  No |
| --- | --- | --- | --- |
| **Title and abstract** | 1 | (*a*) Indicate the study’s design with a commonly used term in the title or the abstract | 1 |
|  |  | (*b*) Provide in the abstract an informative and balanced summary of what was done and what was found | 1 |
| Introduction | | | |
| Background/rationale | 2 | Explain the scientific background and rationale for the investigation being reported | 2 |
| Objectives | 3 | State specific objectives, including any prespecified hypotheses | 2 |
| Methods | | | |
| Study design | 4 | Present key elements of study design early in the paper | 3 |
| Setting | 5 | Describe the setting, locations, and relevant dates, including periods of recruitment, exposure, follow-up, and data collection | 2 |
| Participants | 6 | (*a*) *Cohort study*—Give the eligibility criteria, and the sources and methods of selection of participants. Describe methods of follow-up  *Case-control study*—Give the eligibility criteria, and the sources and methods of case ascertainment and control selection. Give the rationale for the choice of cases and controls  *Cross-sectional study*—Give the eligibility criteria, and the sources and methods of selection of participants | 2 |
|  |  | (*b*) *Cohort study*—For matched studies, give matching criteria and number of exposed and unexposed  *Case-control study*—For matched studies, give matching criteria and the number of controls per case | - |
| Variables | 7 | Clearly define all outcomes, exposures, predictors, potential confounders, and effect modifiers. Give diagnostic criteria, if applicable | 3 |
| Data sources/ measurement | 8* | For each variable of interest, give sources of data and details of methods of assessment (measurement). Describe comparability of assessment methods if there is more than one group | 2 |
| Bias | 9 | Describe any efforts to address potential sources of bias | 4 |
| Study size | 10 | Explain how the study size was arrived at | 2 |
| Quantitative variables | 11 | Explain how quantitative variables were handled in the analyses. If applicable, describe which groupings were chosen and why | 3 |
| Statistical methods | 12 | (*a*) Describe all statistical methods, including those used to control for confounding | 4 |
|  |  | (*b*) Describe any methods used to examine subgroups and interactions | 4 |
|  |  | (*c*) Explain how missing data were addressed | 4 |
|  |  | (*d*) *Cohort study*—If applicable, explain how loss to follow-up was addressed  *Case-control study*—If applicable, explain how matching of cases and controls was addressed  *Cross-sectional study*—If applicable, describe analytical methods taking account of sampling strategy | - |
|  |  | (*e*) Describe any sensitivity analyses |  |

Continued on next page

| Results | | | |
| --- | --- | --- | --- |
| Participants | 13* | (a) Report numbers of individuals at each stage of study—eg numbers potentially eligible, examined for eligibility, confirmed eligible, included in the study, completing follow-up, and analysed | 5 |
|  |  | (b) Give reasons for non-participation at each stage | 5 |
|  |  | (c) Consider use of a flow diagram | 5 & Supplementary Figure 1 |
| Descriptive data | 14* | (a) Give characteristics of study participants (eg demographic, clinical, social) and information on exposures and potential confounders | 5 & Table 1 |
|  |  | (b) Indicate number of participants with missing data for each variable of interest | 5 |
|  |  | (c) *Cohort study*—Summarise follow-up time (eg, average and total amount) | 5 |
| Outcome data | 15* | *Cohort study*—Report numbers of outcome events or summary measures over time | 5-6 |
|  |  | *Case-control study—*Report numbers in each exposure category, or summary measures of exposure | *-* |
|  |  | *Cross-sectional study—*Report numbers of outcome events or summary measures | *-* |
| Main results | 16 | (*a*) Give unadjusted estimates and, if applicable, confounder-adjusted estimates and their precision (eg, 95% confidence interval). Make clear which confounders were adjusted for and why they were included | 5-6 & Table 3 |
|  |  | (*b*) Report category boundaries when continuous variables were categorized | Table 2 |
|  |  | (*c*) If relevant, consider translating estimates of relative risk into absolute risk for a meaningful time period | - |
| Other analyses | 17 | Report other analyses done—eg analyses of subgroups and interactions, and sensitivity analyses | 6 |
| Discussion | | | |
| Key results | 18 | Summarise key results with reference to study objectives | 6 |
| Limitations | 19 | Discuss limitations of the study, taking into account sources of potential bias or imprecision. Discuss both direction and magnitude of any potential bias | 8-9 |
| Interpretation | 20 | Give a cautious overall interpretation of results considering objectives, limitations, multiplicity of analyses, results from similar studies, and other relevant evidence | 9 |
| Generalisability | 21 | Discuss the generalisability (external validity) of the study results | 9 |
| Other information | | | |
| Funding | 22 | Give the source of funding and the role of the funders for the present study and, if applicable, for the original study on which the present article is based | Title page |

*Give information separately for cases and controls in case-control studies and, if applicable, for exposed and unexposed groups in cohort and cross-sectional studies.

**Note:** An Explanation and Elaboration article discusses each checklist item and gives methodological background and published examples of transparent reporting. The STROBE checklist is best used in conjunction with this article (freely available on the Web sites of PLoS Medicine at http://www.plosmedicine.org/, Annals of Internal Medicine at http://www.annals.org/, and Epidemiology at http://www.epidem.com/). Information on the STROBE Initiative is available at www.strobe-statement.org.
